# Mechanism of Base-Catalyzed Resorcinol-Formaldehyde and Phenol-Resorcinol-Formaldehyde Condensation Reactions: A Theoretical Study

**DOI:** 10.3390/polym9090426

**Published:** 2017-09-07

**Authors:** Taohong Li, Ming Cao, Jiankun Liang, Xiaoguang Xie, Guanben Du

**Affiliations:** 1 The Yunnan Province Key Lab of Wood Adhesives and Glued Products, Southwest Forestry University, Kunming 650224, China; caominghappy@swfu.edu.cn (M.C.); liangjiankun@swfu.edu.cn (J.L.);; 2 Key Lab for Forest Resources Conservation and Utilisation in the Southwest Mountains of China, Southwest Forestry University, Ministry of Education, Kunming 650224, China; 3 School of Chemical Science and Technology, Yunnan University, Kunming 650091, China; xgxie@ynu.edu.cn

**Keywords:** resorcinol-formaldehyde, base-catalyzed, condensation, quinonemethide

## Abstract

The base-catalyzed resorcinol-formaldehyde condensation reactions were theoretically investigated in this study by employing a quantum chemistry method. The condensation reaction includes two steps: (1) formation of the quinonemethide (QM) intermediate from hydroxymethylresorcinol; (2) Michael addition between the quinonemethide and resorcinol anion. The first step is the rate-determining step. Two mechanisms, unimolecular elimination of the conjugate base (E1cb) and water-aided elimination (WAE), were identified for the formation of QM. The hydroxymethylresorcinol anion produces neutral QM while the dianion produces a quinonemethide anion (QMA). The calculated potential energy barriers suggested that the QMA formation is much more favorable. Although resorcinol-formaldehyde and phenol-formaldehyde condensations share a common mechanism, the former would be faster if the QMA participates in condensations. The potential energy barriers for formation of 2-QM, 4-QM, 6-QM, 2-QMA, and 4-QMA were calculated. The results show that the formations of 6-QM and 4-QMA have relatively lower energy barriers. This rationalized previous experimental observations that the 2,4-(2,6-) and 6,6′-(4,4′-) methylene linkages were dominant, whereas the 2,2′-linkage was almost absent. The resorcinol-phenol-formaldehyde co-condensations were also calculated. The cold-setting characteristic of phenol-resorcinol-formaldehyde co-condensed resin can be attributed to participation of resorcinol quinonemethides in condensations.

## 1. Introduction

It is known that the base or acid catalyzed resorcinol-formaldehyde (RF) reactions can form polymeric resins which are currently used as wood adhesives and composites [1,2,3,4,5,6,7], forms [8], and organic gels [9,10,11,12]. Depending on the F/R molar ratio, RF reactions can result in novlac or resol type resin [2]. RF resin is generally not used separately as wood adhesive due to its instability and high cost. Alternatively, in order to accelerate the cure of PF resin, resorcinol is generally mixed with PF resol resin, obtaining the so-called phenol-resorcinol-formaldehyde (PRF) co-condensed resins [2,3,5,6,7]. Due to the similarity of chemical structure to phenol, it is natural to believe the RF polymers are also similar to phenol-formaldehyde (PF) polymers in which methylene bridges can be formed though reactions between phenol and hydroxymethylphenol or between hydroxymethylphenols. However, a significant difference between PF and RF resin is that curing of PF resin generally requires a hot-press temperature above 160 °C, whereas RF and PRF are cold-setting resins. This indicates that RF condensations encounter a much lower energy barrier and occur much faster. Superficially, higher reactivity may be attributed to the stronger nucleophilicity of resorcinol since it has two phenolic hydroxyl groups which lead to higher electron density on the aromatic ring. In the viewpoint of a mechanism at the molecular level, formation of reactive intermediates is the key factor that determines the rate of the condensation reaction. Therefore, comparing the kinetics of the PF and RF intermediates formation can help us better understand the different behaviors of the two resins. But, the experimental kinetics study seems to be incapable of doing so because capture and quantitative description of such short-lived intermediates is currently almost impossible, especially for solution reactions. Fortunately, theoretical methods have become powerful tools that allow us to explore the microscopic world of chemical reactions in silico. In the field of wood adhesive resins, pioneering works that employed molecular mechanics methods are from the group of Professor A. Pizzi [13,14,15,16,17]. These studies have investigated the interactions between resin components and wood cellulose, aiming to establish theoretical models for resin–wood surface interaction and giving insight into the relationship between resin structure and performance. The obtained results can help us design novel resins, but the prerequisite is that we can control the reactions based on our knowledge about mechanisms. Therefore, understanding the reaction mechanisms and resin chemistry is another task.

Different from molecular mechanics, which is based on force filed theory and mainly deals with the hydrogen bonding interactions, Van der Waals’ force and their influences on molecule energy and conformations, quantum chemistry methods that is based on quantum mechanics can deal with formation and breakage of chemical bonds, kinetics and thermodynamics of chemical reactions through rigorous calculations on the electronic structures of the involved species. To explain the different condensation reactivity of PF and RF, quantum chemistry calculations appear to be necessary.

**Figure 1:**
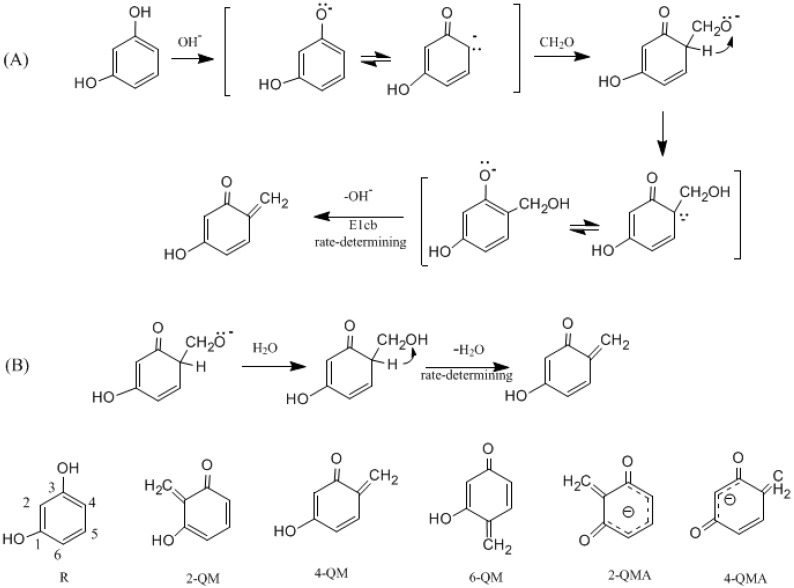
The proposed mechanisms for formation of resorcinol quinonemethides.

By employing a quantum chemistry method, we recently studied the base-catalyzed phenol-formaldehyde reactions and the quinonemethide (QM) was confirmed to be the key intermediate that initiates condensations [18]. RF condensation reactions may share the same mechanism. But, the chemistry of resorcinol is obviously more complex than phenol. Resorcinol has two hydroxyl groups and therefore dissociation of the protons can produce a singly charged or doubly charged anion (dianion) with the presence of a base. As a result, different quinonemethide intermediates may be formed. As can be seen in Figure 1, five possible intermediates may be formed according to the proposed mechanism. 2-QM, 4-QM, and 6-QM are neutral species that are similar to phenol quinonemethides. But, 2-QMA and 4-QMA are anions which may exhibit different reactivity. According to the results for PF reactions [18], formation of quinonemethide is the rate-determining step of the overall condensation reaction. Thus, it can be speculated that formations of RF quinonemethides encounter lower energy barriers. But which of the neutral QMs and QM anions are more favorable? This is the first issue to be addressed in this study.

Another task of this work is to rationalize the experimentally observed condensed structure of RF resin. Theoretically, resorcinol has three reactive sites on the ring, namely, the three *ortho* positions of the two hydroxyl groups. Thus, three types of methylene linkages through 2,2′-, 2,4-, and 4,4′-condensations should be formed. But, it is interesting that some ^13^C NMR studies indicated that the 2,4′- and 4,4′-linkages were found to be dominant, whereas 2,2′-linkage was observed to be minor or absent [2,3,4]. Further, 4,4′-linkage appeared to be more favorable than 2,4-linkage. These observations imply that the 4-position is more reactive than 2-position. A plausible explanation is the 4-position is more reactive toward formaldehyde, leading to higher concentration of 4-hydroxymethylresorcinol. But the hydroxymethylation reaction should be faster than condensations, considering that resorcinol is highly reactive toward formaldehyde in the presence of catalysts. Therefore, different reaction rates of rate-determining steps may be another reason. That is to say, 2-hydroxymethylresorcinol and 4-hydroxymethylresorcinol may have different rates of forming reactive intermediates. Which is the more important factor? Or do both of them work? This issue has never been addressed in detail, according to our survey of the literature.

Finally, in the synthesis of phenol-resorcinol-formaldehyde resins, PF and RF self-condensations and PRF co-condensations should occur simultaneously. The competitive relationship of the reactions is also an important issue that deserves attention because such a relationship determines whether co-condensed structures can be formed efficiently.

## 2. Theoretical Calculations

By employing the density functional theory [19,20] (DFT) method B3LYP [21,22,23] with the standard basis set 6-31++G** [24,25,26,27], full structural optimizations were performed on all the stationary points on the reaction potential energy surfaces (PES), including reactants, intermediates, transition states, and products. The harmonic vibrational frequencies were calculated at the same theoretical level to characterize the nature of the stationary point as a local minimum or first-order saddle point (transition state) on the PES. The zero-point vibrational energies (ZPVE) were used to correct the relative energies. Each transition state (TS) was identified as it has a unique imaginary frequency. Intrinsic reaction coordinate (IRC) analysis was also performed to confirm the connectivity between a TS and a local minimum. To compare the relative nucleophilicity of different carbons of the resorcinol anion and dianion, the APT (atomic polar tensor) charges were also calculated. To simulate the implicit solvent effects, for all the calculations the self-consistent reaction field (SCRF) method was employed with the polarizable continuum model (PCM) [28,29,30] by defining water as the solvent (Water: ε = 78.3553). All the calculations were carried out with the GAUSSIAN 03 program package (Gaussian, Pittsburgh, PA, USA) [31].

The potential energy barrier (theoretical activation energy) of each calculated reaction step was described by the relative energy (energy gap) between a transition state and a reactant or reactant-like intermediate. The relative energies were obtained by the following formulas:

Δ*E*(TS) = [(*E*(TS) + ZPVE(TS)] − [*E*(R) + ZPVE(R)]

Δ*E*(P) = [(*E*(P) + ZPVE(P)] − [*E*(R) + ZPVE(R)]

Here, *E*(TS) is the total electron energy of a TS, while *E*(R) represents the total electron energy of a local minimum which can be an initial reactant or a reactant-like intermediate. Thus, Δ*E*(TS) represents the relative energy between TS and reactant or reactant-like intermediate, namely the energy barrier of a reaction step. Similarly, Δ*E*(P) corresponds to the relative energy between a product or product-like intermediate. In this context, the potential energy profile shown in this paper was demonstrated by choosing the energy of reactant or a reactant-like intermediate as reference.

## 3. Results and Discussion

### 3.1. Formations of Quinine Methide Intermediates

Figure 1 shows two mechanisms for base-catalyzed formation of quinonemethide which was derived from that identified for phenol-formaldehyde reactions [18]. Pathway (A) demonstrates the E1cb (unimolecular elimination of conjugate base) mechanism. Pathway (B) is another one in which intra-molecular water elimination is involved.

According to the proposed mechanisms, three neutral quinonemethide intermediates, 2-QM, 4-QM, and 6-QM, can be produced from singly charged hydroxymethylresorcinol. If a resorcinol dianion participates in the reaction, a quininemethide anion will be formed. Concentrations of the two types of intermediate are dependent on the concentration of the alkaline catalyst (or pH).

**Figure 2:**
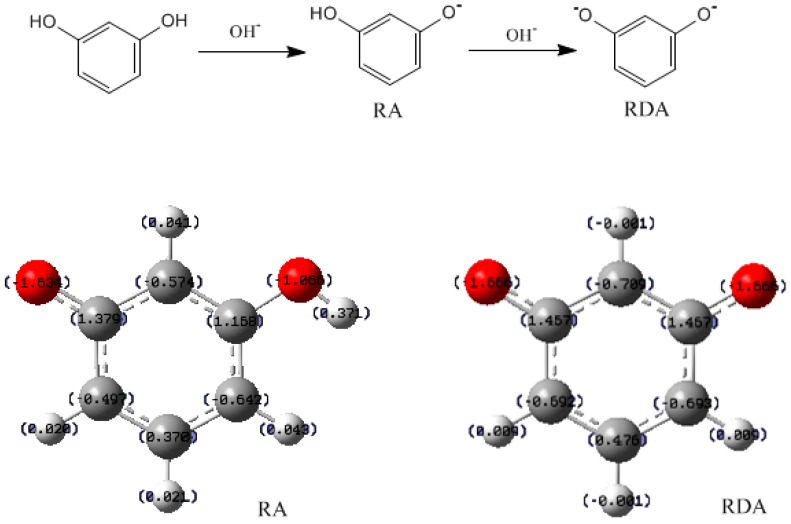
The calculated atomic charges for resorcinol anion (RA) and dianion (RDA).

In the experiment of Christiansen [4], 76% formaldehyde was converted to 4- or 6-hydroxymethyl groups (4- and 6-position are equal due to symmetry of resorcinol), whereas only 12% formaldehyde took the form of 2-hydroxymethyl groups. Three factors may be attributed to this result. First, the 4- and 6-positions are more reactive toward formaldehyde due to their stronger nucleophilicity. Another one is a statistical factor. Namely, two positions versus one. Finally, Durairaj pointed out that the steric hindrance on the 2-position caused by the two adjacent hydroxyl groups is a key factor [32]. To confirm the first factor, the APT atomic charges were calculated and are given in Figure 2. In the resorcinol anion (RA), the carbons numbered as 2, 4, and 6 are negatively charged as −0.574, −0.479, and −0.642, while carbon 5 is positively charged as 0.378, clearly indicating the three *ortho* positions are strong nulceophilic centers. But, according to the charge distribution, the three positions are different in reactivity as the nucleophilic order appears to be 6 > 2 > 4. Position 4 is less reactive than position 2, suggesting that, for RA, high reactivity of 6-position is responsible for dominant formation of corresponding hydroxymethyl groups. In contrast with the statistical factor, nucleophilicity plays a more important role. For the resorcinol dianion (RDA), the negative charges of the three *ortho* positions were almost equal. But, the statistical factor determines 4- and 6-positions have higher probability in reacting with formaldehyde. Steric hindrance may be partially responsible for the slower hydroxymethylation in 2-postion, but it cannot rationalize the results that the 2-hydroxymethyl groups and 2,4′-methylene linkages were produced while 2,2′-methylene linkages were absent. These results also cannot be explained by nucleophilicity and statistical effects. Slower hydroxymethylation is not definitely in accordance with slower condensation reactivity. Theoretical calculations on PF reactions revealed that quinonemethide formation is the rate-determining step in the overall condensation reaction, and formation of *para* quinonemethide is faster than the formation of *ortho* quinonemethide [18]. This explained the experimental results that *ortho*-*ortho*-methylene linkage was always observed to be minor in comparison with *ortho*-*para*- and *para*-*para*-methylene linkages. So, it is also necessary to compare the reactivity of 2-, 4-, and 6-hydroxymethylresorcinol anion (HMRA) in formation of quinonemethide intermediates.

**Figure 3:**
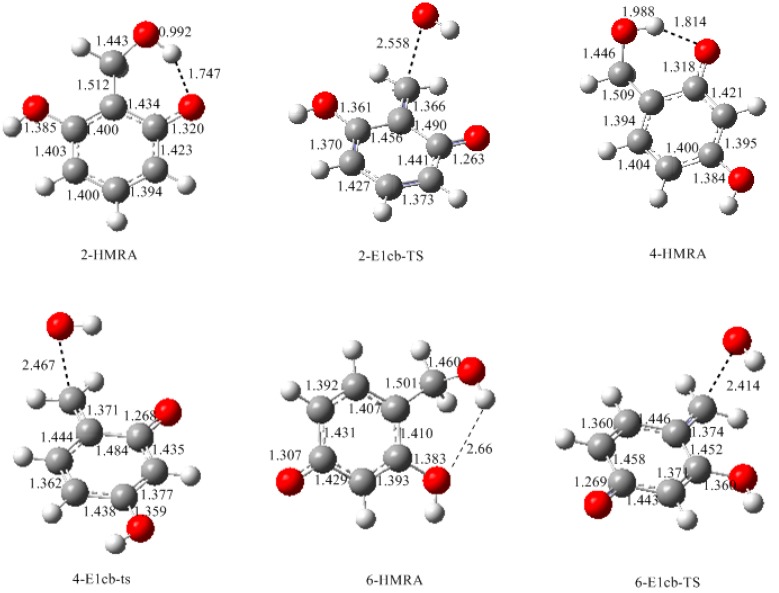
The calculated structures of the intermediates and transition states for E1cb formation of quinonemethide from hydroxymethyresorcinol anion.

**Figure 4:**
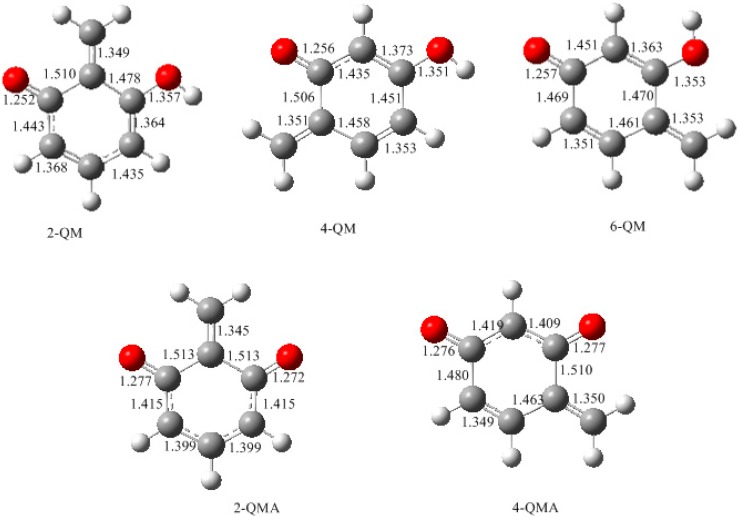
The calculated structures for the neutral quinonemethides (QM) and quinonemethide anions (QMA).

**Figure 5:**
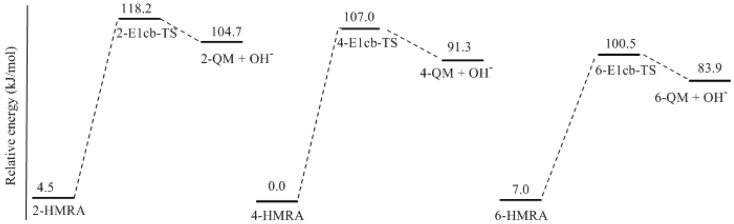
The potential energy profiles for E1cb formations of quinonemethides (QM).

Figure 3 shows the calculated structures of the reactant intermediates and transition states for the E1cb formation of different neutral quinonemethides from hydroxymethylresorcinol anions. The structures of produced quinonemethides are collected in Figure 4. In 2-hydroxymethylresorcinol anion (2-HMRA), a strong intra-molecular hydrogen bond (1.747 Å) was formed between hydroxymethyl group and the carbonyl oxygen. In contrast, the hydrogen bond in 4-hydroxymethylresorcinol anion (4-HMRA) is weaker since the bond length is longer (1.814 Å). In 6-HMRA, the hydrogen bond is formed between two hydroxyl groups and the very long bond length of 2.660 Å indicates the interaction is almost ignorable. The hydrogen bonding effects seems to be a factor that stabilizes these species. But, the bond length order is not consistent with the stability order, as it can be seen that the 4-HMRA is the most stable one, therefore it was chosen as reference in the potential energy profiles. In fact, besides the hydrogen bonding interaction, the electronic structures of the isomers also play an important role, especially the delocalization extent of electrons in the conjugate system. However, as can be seen from the potential energy profiles in Figure 5, the E1cb transition state (2-E1cb-TS) has a higher energy barrier (113.7 kJ/mol) than 4-E1cb-TS (107.3 kJ/mol) by 6.7 kJ/mol. Further, 6-E1cb-TS has the lowest energy barrier of 93.5 kJ/mol. This order is consistent with the order of hydrogen bonding strength. Obviously, the hydrogen bonding effect suppresses the leaving of an OH ion and the relative bonding strength is the key factor that determines the relative reactivity for HMRAs to form quinonemethide intermediates. In our calculations for PF reactions [18], such effect was also found. In addition, the quinonemethides, 2-QM, 4-QM, and 6-QM are different in stability. Specifically, 6-QM is the most stable species and is more stable than 2-QM and 4-QM by 20.8 and 7.4 kJ/mol, respectively. Thus, considering both the energy barriers and relative stability of different quinonemethides, formation of 6-QM is energetically the most favorable and condensations involving 6-QM are the fastest, whereas condensations related to 2-QM are less competitive. The reaction of 6-QM with another monomer, resorcinol or hydroxymethylresorcinol, will lead to 2-6 or 4-6 methylene linkage. Due to the symmetric structure of resorcinol, they are equal to 2,4 or 4,4′-methylene linkage. Therefore, in addition to the higher concentration of 4- or 6-hydroxymethyl group, the lower energy barrier for 6-QM formation also plays an important role. Correspondingly, because of the lower concentration of the 2-hydroxymethyl group and higher energy barrier for formation of 2-QM, 2,2′-methylene linkage was almost prohibited, or only very minorly formed.

**Figure 6:**
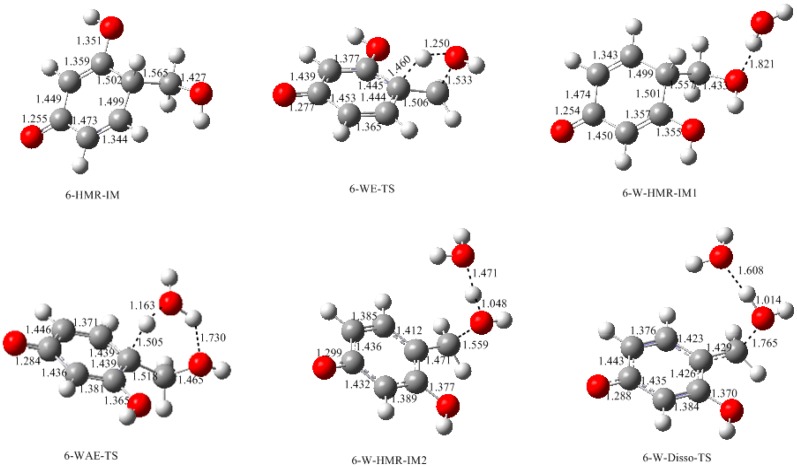
The calculated structures of the intermediates and transition states for the formation of 6-QM (quinonemethide) via the WE (water elimination) and WAE (water-aided elimination) mechanisms.

**Figure 7:**
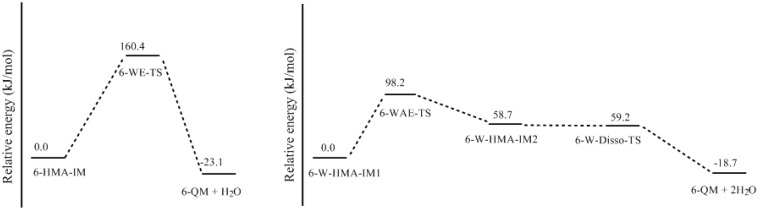
The potential energy profiles for formation of 6-QM via the WE and WAE mechanisms.

In our recent calculations on PF condensation reactions [18], a new mechanism which involves intra-molecular water elimination was identified. For RF, such a mechanism was shown in Figure 1 as pathway (B). Here, only the reaction on carbon 6 was considered as an example. The calculated results are given in Figure 6 and Figure 7. Note that the 6-HMRA (Figure 3) is an anion, while the intermediate (IM) 6-HMR-IM is a neutral species. Starting from this reactant-like intermediate, a water molecule can be eliminated (WE) via the four-member ring transition state WE-TS, leading to 6-QM. This reaction encounters a notable barrier of 160.4 kJ/mol, indicating that such a mechanism cannot compete with E1cb at all. However, for PF, it was found that solvent water molecules can catalyze the proton transfer from carbon to oxygen and the energy barrier was significantly lowered [13]. We call such a mechanism a water-aided elimination (WAE). It was found that such a mechanism can also be applied to RF. 6-W-HMR-IM1 is the complex formed through inter-molecular hydrogen bonding between 6-HMR and a water molecule. Via the six-member ring transition state WAE-TS, the proton on carbon 6 migrates to water molecule. Meanwhile, a proton on water shifts to the oxygen atom of the hydroxymethyl group. Consequently, the product-like intermediate 6-W-HMR-IM2 is formed. WAE-TS has energy barrier of 98.2 kJ/mol which is significantly lower than WE-TS by 62.2 kJ/mol, indicating that solvent water has a very strong catalytic effect. Elimination of a two-water complex via 6-Disso-TS finally leads to 6-QM. This step is almost barrierless. The barrier of WAE is slightly higher than that of E1cb (6-E1cb-TS) by 4.7 kJ/mol, implying that such a mechanism may also contribute to the formation of 6-QM. Differently, for PF, the WAE mechanism was found to be energetically more favorable than the E1cb mechanism [18]. Specifically, the barrier of the WAE reaction on the *para* position was found to be 8.0 kJ/mol lower than that of the E1cb reaction. This difference suggests that the WAE and E1cb mechanisms play different roles for PF and RF systems.

**Figure 8:**
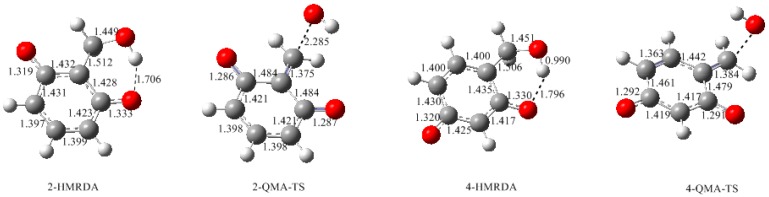
The calculated structures for the intermediates and transition states for the E1cb formation of quinonemethide anions.

**Figure 9:**
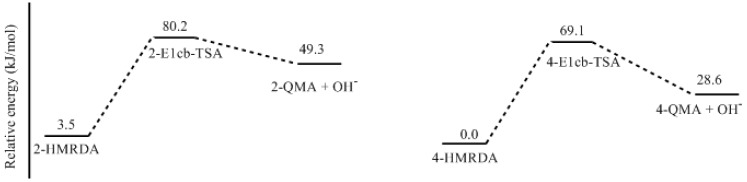
The potential energy profiles for the E1cb formation of quinonemethide anions (QMAa).

Dissociation of the two phenolic hydroxyl groups of resorcinol leads to a dianion which should be a stronger nucleophile. Therefore, hydroxymethylation of such a dianion can produce hydroxymethylresorcinol dianion (HMRDA), from which a quinonemethide intermediate can also be formed. According to the proposed mechanism in Figure 1, the formed quinonemethides should be anions like 2-QMA and 4-QMA. The calculated results for the formation of them are shown in Figure 8 and Figure 9. The transition states 2-E1cb-TSA and 4-E1cb-TSA have energy barriers of 76.7 and 69.1 kJ/mol, which are significantly lower than 2-E1cb-TS and 4-E1cb-TS by 37.0 and 24.4 kJ/mol, respectively. Therefore, condensations related to 2-QMA and 4-QMA should be much faster. Similarly, formation of 4-QMA is energetically more favorable than 2-QMA. However, as resorcinol is a weak acid, the concentrations of resorcinol dianion (RDA) and hydroxymethylresorcinol dianion (HMRDA) are dependent on the concentration of the base or the base/resorcinol molar ratio.

### 3.2. RF and PRF Polyaddtion Reactions

**Figure 10:**
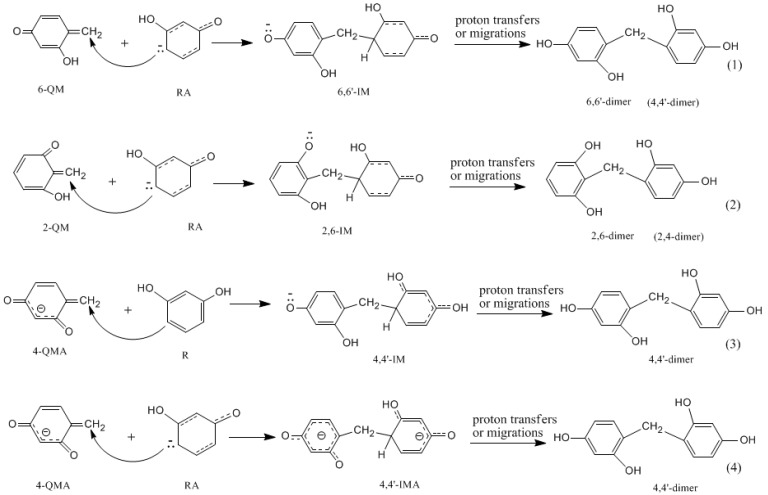
The selected Michael addition reactions of resorcinol anion (RA) and neutral resorcinol (R) with quinonemethides.

**Figure 11:**
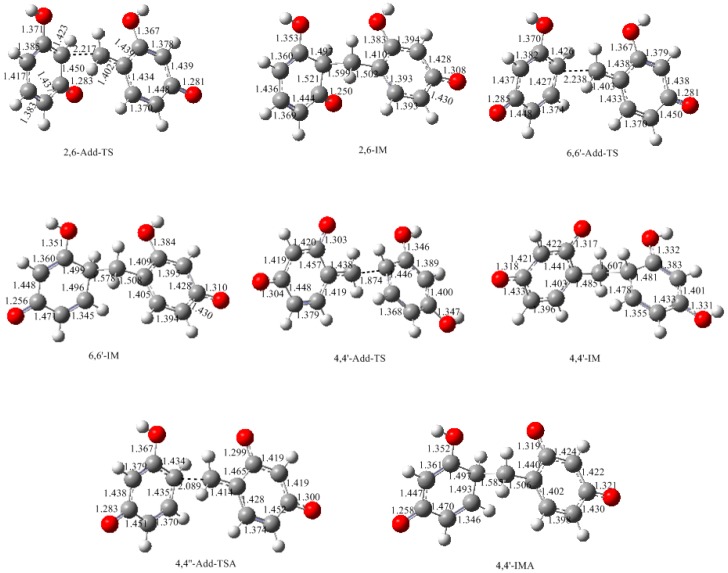
The calculated structures of transition states and product-like intermediates for the Michael addition reactions in Figure 10.

**Figure 12:**
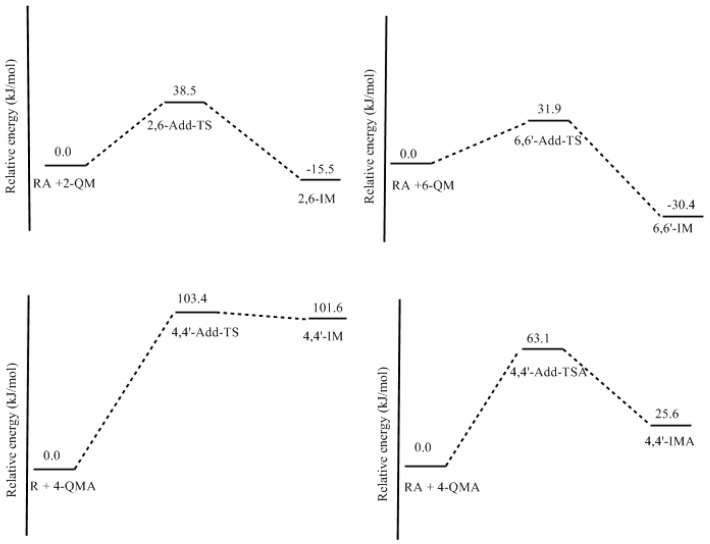
The potential energy profiles for the Michael addition reactions in Figure 10.

As it was found for PF reactions [18], once the quinonemethide is formed, a Michael addition reaction will take place between a neutral quinonemethide and a phenol anion or a hydroxymethylphenol anion. Therefore, “polyaddition” may be a more appropriate word than “polycondensation” to describe the reaction from a mechanistic viewpoint. Such a reaction was identified to be much faster than quinonemethide formation. This feature should still be kept for neutral RF quinonemethides. But does it still hold true for a quinonemethide anion, like 4-QMA? It is expected that the reaction between two anions (or electron-rich species) would encounter a repulsive effect. Then, what if the condensation occurs between a quinonemethide anion and a neutral monomer? To answer these questions, the condensation reactions shown in Figure 10 were calculated and the results are shown in Figure 11 and Figure 12.

Reactions (1) and (2) are the reactions of neutral 2-QM and 6-QM with a resorcinol anion. Theoretical calculations predicted that they have energy barriers of 38.5 and 31.9 kJ/mol, represented by 2,6-Add-TS and 6,6′-Add-TS, respectively, which are much lower than those for formations of the quinonemethides. Therefore, they are indeed faster reactions. As the formation of quinonemethide is the rate-determining step, the overall condensation reaction can be identified as having a unimolecular pattern. The formed intermediate products 6,6′-IM and 2,6-IM can evolve to 6-6′-dimer (4,4′-dimer) and 2,6-dimer (2,4-dimer), respectively. Several steps of proton transfers or migrations may occur in this process, but they are generally fast reactions. Another reaction leading to the 2,4-dimer is the one between 4-QM or 6-QM and the 2-position of the resorcinol anion. But, the situation should be similar to reaction (2).

Differently, reaction (3), the condensation of 4-QMA with neutral resorcinol encounters a notable barrier of 103.4 kJ/mol which significantly exceeded the barrier of 4-QMA formation (69.1 kJ/mol) and became the rate-determining step. This must be caused by the much lower nucleophilic reactivity of neutral resorcinol. It was not our expectation that reaction (4) would encounter a much lower barrier of 63.1 kJ/mol. Although the repulsive effect makes the barrier higher than that of reactions (1) and (2), the barrier is still lower than that of the formation of 4-QMA (69.1 kJ/mol). Therefore, such a condensation pattern is feasible. But, the overall reaction may not be a typical unimolecular mechanism because the 4-QMA formation and following Michael addition have similar energy barriers. In fact, 4-QMA may not directly participate in the condensation because it can convert to 4-QM or 6-QM through fast protonation of one of the carbonyl groups. Thus, the pathway with the lowest energy barrier should be 4-HMRDA→4-QMA→protonation→4-QM (or 6-QM).

So far, there is no experiment that compares PF and RF condensation reaction rates under the same conditions (the same F/P or F/R molar ratio, catalyst concentration, and temperature) that has been reported. But, the calculated potential energy barriers for PF and RF quinonemethides allow us to make comparisons since the formation of these intermediates is the rate-determining step. According to our theoretical calculations of PF [18], the lowest energy barrier for E1cb formation of neutral *para*-quinonemethide was calculated to be 99.7 kJ/mol, which is higher than that of 6-QM (93.5 kJ/mol) by 6.2 kJ/mol, but the WAE mechanism for PF has a lower barrier of 87.8 kJ/mol. In this sense, RF condensations are not faster. But, taking into account the participation of the quinonemethidesanions like 4-QMA, RF condensations should be much faster since the energy barrier for its formation is significantly lower than the neutral PF QMs by 20–30 kJ/mol. Such a big difference is sufficient to explain the fact that the RF resin cure is much faster than PF resin, and that RF resin can be used as PF cure accelerator.

**Figure 13:**
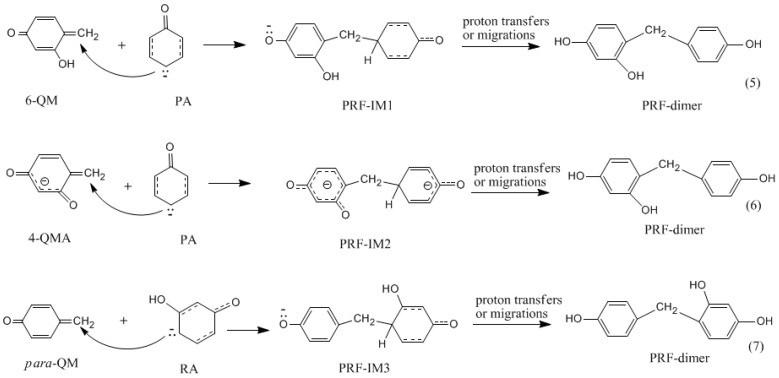
The Michael addition reactions of phenol anion (PA) with resorcinol quinonemethides (6-QM and 4-QMA) and the reaction of resorcinol anion (RA) with phenol quinonemethide (*para*-QM).

**Figure 14:**
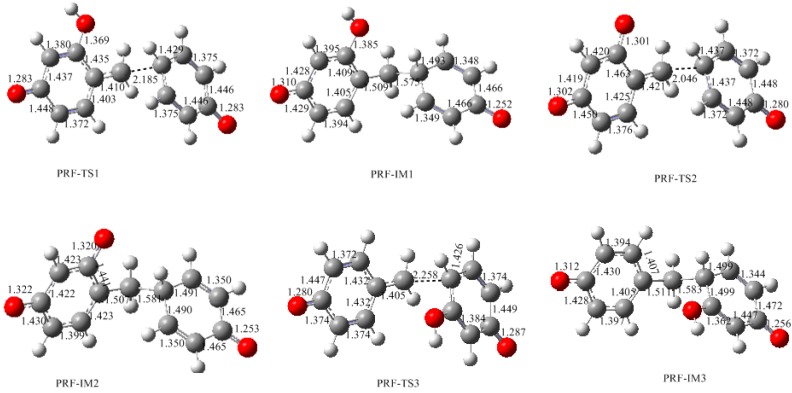
The calculated structures of the transition states and product-like intermediates for the Michael addition reactions in Figure 13.

**Figure 15:**
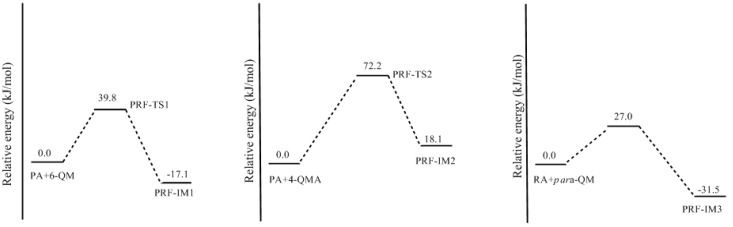
The potential energy profiles for the Michael addition reactions in Figure 13.

Synthesis of phenol-resorcinol-formaldehyde (PRF) co-condensed resin has been a hot topic [1,2,3,5,6,8]. In preparation of traditional phenol-resorcinol-formaldehyde resin, resorcinol is generally added into the PF resol resin. It is believed that resorcinol can react with the hydroxymethyl groups of PF resin, forming P–CH_2_–R methylene linkages [3]. With such a pattern, resorcinol can be connected to the end of PF chains. Conversely, can phenol react with the hydroxymethyl group on resorcinol? What will happen if phenol, resorcinol, and formaldehyde are mixed together? How do co-condensations compete with self-condensations? These issues have never been addressed mechanistically in detail. According to our understanding, the co-condensation efficiency is not only dependent on the rates of PF and RF quinonemethides formation, but also on the selectivity of these intermediates toward PF and RF monomers. Namely, both PF and RF quinonemethide intermediates can be formed under alkaline conditions and their relative formation rates and reactivity toward phenol, resorcinol, and their hydroxymethyl compounds determines the competitive formations of self- and co-condensed polymers. To address this issue, we carried out further calculations on the representative PRF co-polyaddition reactions which are shown in Figure 13 as reactions (5)–(7). The calculated results are given in Figure 14 and Figure 15.

Reaction (5) is the addition between the phenol anion and resorcinol quinonemethide (6-QM). The calculated energy barrier for this reaction is 39.8 kJ/mol, which is higher than reaction (1) by 7.9 kJ/mol, and higher than the addition of the phenol anion with phenol quinonemethide by 13.2 kJ/mol [18]. This result indicates that the co-condensation reaction between phenol and hydroxymethylresorcinol is less competitive than PF and RF self-condensations. Similarly, reaction (6) encounters a higher barrier than self-condensations. In contrast, reaction (7) has the lowest barrier of 27.0 kJ/mol, which is lower than the barrier of reaction (1) by 12.8 kJ/mol and very close to the barrier of PF self-condensation (26.6 kJ/mol), suggesting that the co-condensation between the resorcinol anion and phenol quinonemethide is energetically more competitive. In this context, if resorcinol, phenol, and formaldehyde are mixed together, hydroxymethylation of resorcinol should be faster than hydroxymethylation of phenol due to higher reactivity of resorcinol. In addition to faster formation of resorcinol quinonemethides, RF self-condensation occurs prior to PRF co-condensation, but it is unlikely that the PRF reactions can be excluded since the gap between the energy barriers are moderate.

As mentioned in the literature [3], a general strategy in the synthesis of PRF is to let resorcinol react with hydroxymethylphenol or with PF resin in excess of un-reacted hydroxymethyl groups. In such a type of resin, the polymers are resorcinol terminated. In the curing process, condensations can occur between terminal resorcinol moieties, or between phenol and hydroxymethylatedresorcinol. In both cases, fast formation of the resorcinol quinonemethides can contribute to the curing condensations. This is why PRF resin bears the cold-setting characteristic.

## 4. Conclusions

Base-catalyzed resorcinol-formaldehyde condensation reactions were theoretically investigated in this study by employing a quantum chemistry method. The experimentally observed polymer structures were rationalized according to the identified mechanisms and the calculated energetics. The results were discussed and compared with those obtained previously for phenol-formaldehyde reactions. The main conclusions have been drawn as follows:

(1) The condensation reaction includes two steps: Formation of a quinonemethide (QM) intermediate from hydroxymethylresorcinol, followed by Michael addition between the quinonemethide and the resorcinol anion.
(2) Two mechanisms, unimolecular elimination of conjugate base (E1cb) and water-aided elimination (WAE), were identified for the formation of quinonemethide. Hydroxymethylresorcinol anion produces a neutral quinonemethide while the dianion produces a quinonemethide anion. The calculated potential energy barriers suggested that quinonemethide anion formation is much more favorable. Although resorcinol-formaldehyde and phenol-formaldehyde share common condensation mechanisms, the former would be faster if the quinonemethide anion is involved.
(3) The potential energy barriers for formation of 2-QM, 4-QM, 6-QM, 2-QMA, and 4-QMA were calculated. The results show that the formation of 6-QM and 4-QMA have relatively lower energy barriers. In addition to higher reactivity of the position-6 hydroxymethylation reaction, the previous experimental observation that the 2,4-(2,6-) and 6,6′-(4,4′-) methylene linkages were dominant polycondensed structures has been rationalized.
(4) The cold-setting characteristic of phenol-resorcinol-formaldehyde co-condensed resin can be attributed to participation of resorcinol quinonemethides in condensations.
